# Early Detection of Freeze Damage in Navelate Oranges with Electrochemical Impedance Spectroscopy

**DOI:** 10.3390/s18124503

**Published:** 2018-12-19

**Authors:** Emma Serrano-Pallicer, Marta Muñoz-Albero, Clara Pérez-Fuster, Rafael Masot Peris, Nicolás Laguarda-Miró

**Affiliations:** 1 Escuela Técnica Superior de Ingeniería del Diseño (ETSID), Universitat Politècnica de València, Camí de Vera s/n, 46022 Valencia, Spain; emserpal@etsid.upv.es (E.S.-P); marmuoal@etsid.upv.es (M.M.-A.); ramape@eln.upv.es (R.M.P.); 2 Instituto Interuniversitario de Investigación de Reconocimiento Molecular y Desarrollo Tecnológico (IDM), Unidad Mixta Universitat Politècnica de València-Universitat de València, Camí de Vera s/n, 46022 Valencia, Spain; cperezf@eln.upv.es

**Keywords:** orange, freeze damage, detection, sensor, electrochemical impedance spectroscopy

## Abstract

The early detection of freeze damage in Navelate oranges (Citrus sinensis L. Osbeck) was studied using electrochemical impedance spectroscopy (EIS), which is associated with a specific double-needle sensor. The objective was to identify this problem early in order to help to determine when a freeze phenomenon occurs. Thus, we selected a set of Navelate oranges without external defects, belonging to the same batch. Next, an intense cold process was simulated to analyze the oranges before and after freezing. The results of the spectroscopy analysis revealed different signals for oranges depending on whether they had experienced freezing or not. Principal Component Analysis (PCA) and Partial Least Squares-Discriminant Analysis (PLS-DA) of the obtained data demonstrated that it is possible to discriminate the samples, explaining 88.5% of the total variability (PCA) and being able to design a mathematical model with a prediction sensitivity of 80% (PLS-DA). Additionally, a designed artificial neural network (ANN) prediction model managed to correctly classify 100% of the studied samples. Therefore, EIS together with ANN-based data treatment is proposed as a viable alternative to the traditional techniques for the early detection of freeze damage in oranges.

## 1. Introduction

Citrus fruits are one of the most commercialized fruits around the world, with an overall production of more than 124 million tons in 2016, of which almost 67 million correspond to orange production. Spain, with a total production of 6.88 million tons and 3.64 million tons of oranges, is the fifth-largest producing country worldwide [1]. In the current framework regarding the cultivation and commercialization of citrus fruits, the problem of freeze damage to the fruit in the face of intense cold phenomena, either in the countryside when the fruit is still on the tree or due to inadequate refrigerated storage, results in the loss of quality and potential commercialization [2,3]. This problem is potentially more likely to occur in late orange varieties, such as the chosen Navelate variety, which remain on the trees during the entire winter season.

Meteorologically, it is understood that frost occurs when the environment temperature measured under cover drops below 0 °C. However, from an agricultural point of view, frost occurs when the air temperature drops to that point and lasts long enough to damage tissues of plants and fruit due to the formation of ice crystals [4].

In fact, frost affects citrus fruits in very different ways depending on factors such as the intensity of the phenomenon, the type of citrus fruit and its characteristics, the age of the plants, their physiological state, the location, moment and duration of the frost, etc. When these phenomena occur, the economic consequences can be very significant, ranging from slight reductions in productivity to the complete loss of the harvest [5,6].

Currently, there are several techniques to detect freeze damage in fruits. Although the most common ones are the visual observation of pitting on the skin of citrus fruits and the cutting and inspection of the inner part of a fruit sample [7], there are also techniques such as the separation by flotation (density) [8], techniques based on vision and weight sensors [9], fluorescence [3], gas chromatography–mass spectrometry [10], ethanol detection [2], or even nuclear magnetic resonance (NMR) [11]. Nevertheless, these are laboratory methods that are generally complex, time consuming, and expensive and that require specialized personnel as well as a field sampling to be analyzed later in the laboratory. On the contrary, EIS is simple and immediate, does not require reagents or toxic compounds, and is also comparatively inexpensive. In the field of food technology, EIS has already been used in the evaluation of the physiological properties of kiwi fruit [12], the ripening of mango [13], the analysis of eggplant pulp [14], the differentiation between fresh and frozen-thawed sea bream [15], the control of properties in carrots [16,17], the analysis of the effect of temperature on potatoes [18], the study of the behavior of tomato skin [19], the use of pineapple waste for bioethanol production [20,21], and even in the control of steel corrosion by adding orange extracts [22].

In order to conduct an adequate treatment of the huge amount of data generated by EIS assays and to obtain robust and reliable results, a sufficiently powerful mathematical and statistical tool is needed. The analysis of principal components (PCA) and partial least squares (PLS) have proved to be highly valuable mathematical tools for the treatment of this type of data [23,24]. Specifically, a discriminant analysis by partial least squares (PLS-DA) was used in this study, as it is an ideal tool to create classification models when the analyzed samples are associated with a large amount of data [25]. Artificial neural networks (ANNs) also stand out in this field, as they have been widely used to design sample classification systems [26,27]. In fact, ANNs surpass the aforementioned methods due to their enormous flexibility, adaptability, precision to adjust to non-linear systems, and ability to learn from their own mistakes. In addition, classification and modeling systems designed with ANN are clear, simple to use, have low computational requirements, and their algorithms are easily implemented using a PC or a microprocessor. The potential applications of ANNs are of particular interest for this study, since they allow the design of systems for portable devices that can perform on-site analyses with great flexibility, a wide range of applications, easy handling, and low energy requirements. For this type of application [28], other authors have developed simplified ANNs [29,30] reducing the architecture of the networks as well as the equations in their nodes to also reduce the computational requirements. Thereby, the system has become computationally simple, faster to program, and easier to manage, while offering practically the same reliability [31].

Preliminary studies carried out in our research group have allowed us to identify a difference in response between oranges before and after being submitted to a cooling process below its critical freezing temperature. Based on the research described, the objective of this study was to determine the validity of EIS, combined with an appropriate data processing system (the designed ANN), as a technique for the early detection of Navelate oranges that have suffered damage by frost phenomena.

## 2. Materials and Methods

### 2.1. Electrochemical Impedance Spectroscopy System

The EIS technique allows evaluation of the impedance of materials by applying alternate electric signals with different frequencies to them and measuring the corresponding output signals. Impedance can be related to conductivity and other physicochemical parameters, and even to the structure of the samples.

The electronic measuring system was developed by the Group of Electronic Development and Printed Sensors (GED+PS) of the Interuniversity Research Institute for Molecular Recognition and Technological Development (IDM) at the Universitat Politècnica de València (UPV), and it has been described in detail in a previous paper [32]. Essentially, it consists of a device and a software application (interactive graphical interface) that run on a PC. The software application sends the parameters established by the user (e.g., signal amplitude, frequency range, etc.) to the equipment. The device generates electrical signals with a maximum voltage of 500 mV in a frequency range of 1 Hz to 1 MHz over the samples. Then the electronic equipment carries out the measurement of the current and sends the data to the PC. For each analyzed frequency, the system calculates 256 points corresponding to the temporary evolution of the signal in a sinusoidal form, which is immediately drawn and shown on the computer screen. In the designed frequency range, up to 50 different frequencies per assay are analyzed. Finally, the PC calculates the impedance module and phase by means of the discrete Fourier transform (DFT) of the measured current and voltage. The result of the measurement is stored in a file and the data obtained can be plotted in two graphs—the module and the phase plot—where the values of the impedance module and phase, respectively, are plotted versus the frequency.

**Figure 1:**
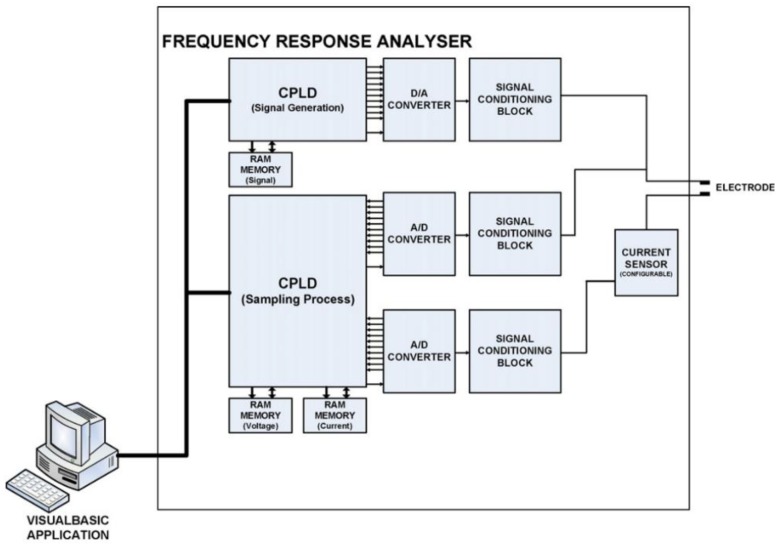
System block diagram of the electrochemical impedance spectroscopy (EIS) device [32]. A/D: analogue-to-digital; D/A: digital-to-analogue.

The device and the computer (Figure 1) are connected through an RS-232 port. The device includes a digital block, a 10-bit digital–analogue converter (DAC), two 8-bit analogue–digital converters (ADCs), and several analogue signal adaption circuits. The digital block includes two logic complex programmable devices (CPLD, Altera EPM7160SLC84, San Jose, CA, USA) and three 2-kB static RAM memories. The first CPLD and one of the RAM memories are in charge of data reception from the PC and of signal generation. An internal universal asynchronous receiver-transmitter (UART) interface lets the CPLD receive and store data in the external RAM. When all the data corresponding to the signal are received, the CPLD outputs them to the DAC converter at a rate that fulfills the signal frequency requirements. The CPLD keeps on repeating the stored pattern until it receives an order to stop from the PC. The DAC output signal is adjusted in voltage, amplified in current, and applied to the electrode. At the same time, a second CPLD samples the signals corresponding to the voltage and current in the electrode. Samples are then stored in the above-mentioned static RAM memories. After sampling a complete cycle of the signal, data are sent to and stored on the PC. Additionally, signal adaption blocks are implemented with wide-bandwidth operational amplifiers. An auto-balancing bridge configuration was used to reduce the effects of high frequencies in the measurement. CPLDs work at a frequency of 25 MHz, thus the maximum frequency for the signal generation and sampling is 25,000,000 points per second. Hence, sinusoidal signals of 1 MHZ are implemented at 25 points per cycle [32].

**Figure 2:**
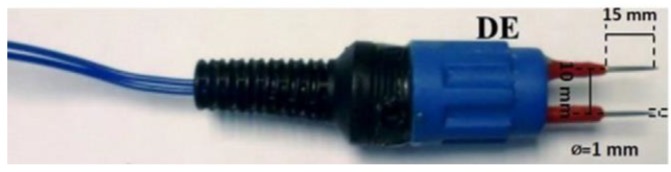
Stainless steel double-needle sensor [33].

EIS measurements can be carried out with two, three, or four electrodes in different configurations. In our case, the measurements were made with two electrodes. In order to detect changes in the internal structure of the samples due to the effects produced by freezing, the GED+PS developed a double-electrode sensor (DE). This sensor consists of two parallel stainless steel needles, an epoxy resin frame as a support, from which the electric cable and the connector exit. The electrodes are 1 mm in diameter, 15 mm long, and 10 mm apart to create a stable electric field. One of the electrodes acts as working electrode and the other as counter electrode (Figure 2). The distance between and the length of the electrodes were designed according to the nature of the samples in order to avoid saturated responses and to obtain a good signal-to-noise ratio.

### 2.2. Laboratory Assays

Navelate oranges were purchased from a local market. First, the fruits to be tested were selected by choosing those with similar characteristics, such as belonging to the same variety, origin, and batch; being of similar caliber and ripeness; and absence of injuries. Of these, a set of ten similar fruits was selected and immediately washed, dried, and stored in a laboratory refrigerator (AQUALYTIC TC-135 S, AQUALYTIC®, Drotmund, Germany) at 13.6 °C until the assays were carried out. 

The laboratory procedure to analyze the samples was rigorously defined before the assays began so that any researcher could replicate the process employed throughout the study. First, the samples were placed on a laboratory table in order to reach room temperature. Then the oranges and the sensor were both cleaned with distilled water and dried with a tissue. Once they were clean, the EIS measurements were conducted. Overall, 27 measurements per sample were carried out in the following way: (a) three measurements per orange inserting the sensor into the skin, taking into account the effect of the peel on the EIS signal; (b) three measurements after having previously removed the skin, to measure EIS signals in only one segment of the orange; and (c) three final measurements between two segments, to consider the effect of the segment membrane. Three iterations of each assay were performed. The EIS measurements were carried out by completely inserting the sensor needles into the fruit and always maintaining the electrodes perpendicular to the fruit surface. All measurements were made at room temperature. Moreover, the sensor was removed, cleaned, dried, and reinserted into the sample between each measurement.

Once the assays were completed, the oranges were placed into a freezer (LIEBHERR Model GGU 1500 Premium, Liebherr-International Deutschland GmbH, Biberach an der Riß, Germany) long enough to assure freezing. For the selected samples, it was enough to keep them at −9 °C for 6 h to reach temperatures below their freezing threshold. After this, the oranges were stored at room temperature to thaw. When room temperature was reached, the EIS measurement procedure, that is, 27 measurements per sample and the corresponding sensor cleaning processes, was repeated. Thus, all the necessary information for the 10 oranges assayed was obtained before and after undergoing the freezing process.

### 2.3. Multivariate Analyses

The objective of the multivariate analysis tools is to establish a statistical prediction model by correlating impedance values obtained by the electronic measurement system and the physical parameters of the samples. First, aPCA analysis was carried out in order to see if the nature of the impedance data of the orange samples that underwent the freezing process differed from that of the rest of the samples. That is, the presence of spontaneous groupings between the measurements was analyzed. Basically, the PCA projects all the multidimensional variability of the samples in a new frame of coordinates composed of two orthogonal directions, maximizing the variance of the input data. PCA also allows the identification of unusual variations within the model, which may indicate the presence of outliers or potential erroneous measurements.

Secondly, a PLS-DA was carried out. The purpose of PLS-DA is to detect significant differences between groups of objects with respect to a set of variables. In fact, it can be considered as a regression analysis with a categorical dependent variable, which can be stated as the label of the group, and a number of continuous independent variables, determining to which groups the samples belong. Therefore, asPCA, it is a classification method. PLS-DA analysis is used to conduct a mathematical description of samples by their distribution in groups and to classify new samples into the previously established groups [34,35].

In this work, both PCA and PLS-DA studies were carried out using amplitude and phase data of 50 impedance measures per assay, logarithmically distributed in the range of 1 Hz to 1 MHz. Regarding the applied pre-processing methods, data were previously autoscaled by means of standardization and mean centering, and a Venetian blinds algorithm was used for the cross-validation.

### 2.4. ANN Modeling

In order to carry out a more precise, flexible, and adaptive prediction model than those traditionally conducted by PLS [35,36], an ANN model was developed. To do so, the software Alyuda Neurointelligence 2.2 © (Alyuda Research Inc., Cupertino, CA, USA) was used. Several preliminary trials allowed us to determine the type and structure of the ANN to be used. Once the type and structure of the ANN were defined, more in-depth work with the software helped us to determine the specific architecture (i.e., number of layers and neurons in each layer), the kinds of algorithms to work with in the layers, and the kinds of functions to be applied in the neurons. 

Data were randomly divided into three sets in order to conduct training (70% of the data), validation (15%), and test (15%) phases. Thus, the training phase allowed the creation of the appropriate ANN model, and the validation and test phases permitted the evaluation of the model by using both previously used data and independent data. As the goal was to conduct a classification ANN, the accuracy of the model was expressed by the correct classification rate (CCR%) and the confusion matrix, clearly indicating the quality of the model by showing the number of elements correctly and incorrectly classified. Finally, the proportional structure of the selected architecture, the use of cross-validation, and the early stopping when training allowed us to avoid overfitting [37].

## 3. Results

### 3.1. Electrochemical Impedance Spectroscopy Results

**Figure 3:**
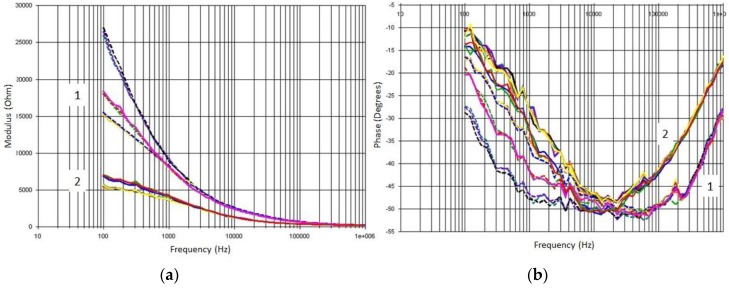
Biplot for both (**a**) modulus and (**b**) phase of the obtained EIS raw data for orange No 1 before (1) and after (2) freezing.

A preliminary configuration of the software allowed us to obtain up to 100 data analyses per assay, that is, 50 analyses of module data and 50 analyses of phase data, which were appropriately saved in a data file for further treatment and graphically represented as shown in Figure 3. The results of the preliminary comparison of EIS data among the assays conducted (a) with the skin (b) without the skin, and (c) without the skin and between two segments showed that we clearly achieved better results in the case of working without the skin and between two segments. Consequently, this section will only refer to the obtained results, discrimination, and modeling for these specific data.

Raw data from these EIS measurements showed a clear difference between signals, depending on whether the sample had been previously frozen of not. These differences were particularly remarkable for modulus data in the range of 100 Hz–10 kHz and for phase data in the range of 100 kHz–1 MHz, clearly indicating that it was possible to identify freeze damage in Navelate oranges via EIS measurements. These differences can be explained from a biological point of view, as the cell wall is a natural insulator acting as a capacitor [38]. The freezing–thawing process destroys an important number of cellular walls that would otherwise remain complete. Thus, broken cells provoke the increase of electrolytes in the orange tissues and also increase the electrical conductivity of the samples. This results in an important decrease of the impedance modulus in the described frequency range. Additionally, this phenomenon produces a diminution of the capacitive behavior of the orange tissue that can be observed in the decrease of the absolute value of the phase in the abovementioned range (Figure 3).

### 3.2. PCA

**Figure 4:**
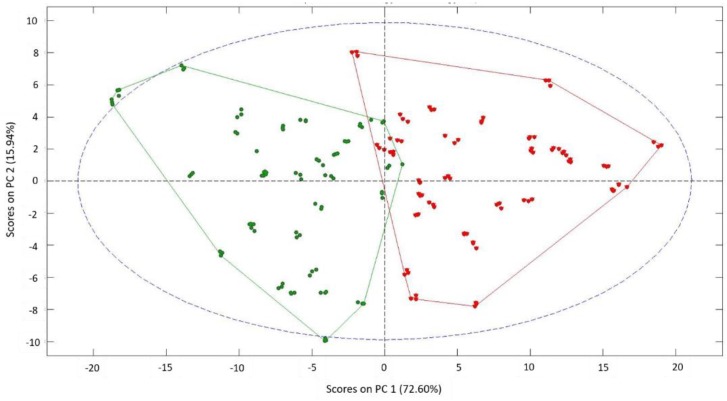
Principal components analysis (PCA) biplot for EIS raw data from the analyzed orange samples: green triangles represent natural (i.e., unfrozen) samples, and red triangles represent those having experienced a freeze phenomenon.

A preliminary PCAcarried out on the results obtained in the EIS tests allowed us to differentiate behaviors of the samples according to whether they had been previously frozen or not. With this PCA analysis, it was possible to explain 72.6% of the total variability of the samples with the first main component (PC1) and an additional 15.9% with the second component (PC2), reaching a total of 88.5% of the total variability being explained with only two main components (Figure 4). Consequently, PC1 can be associated with the freezing phenomenon and PC2 with other variables affecting natural samples such as the studied Navelate oranges.

### 3.3. PLS-DA Analysis

**Figure 5:**
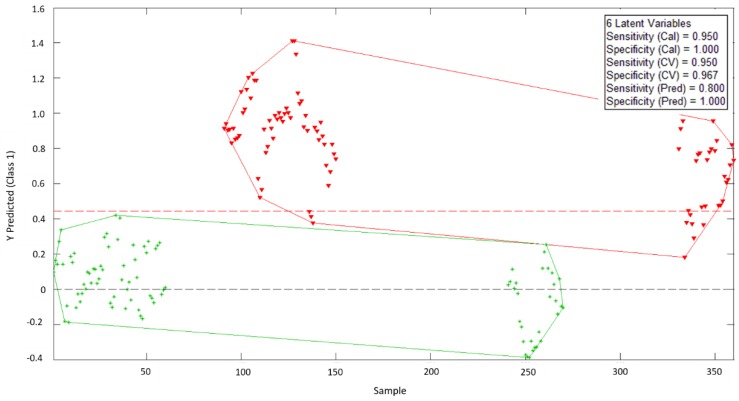
Partial least squares discriminant analysis (PLS-DA) biplot for EIS raw data from the analyzed orange samples: green crosses represent natural (i.e., unfrozen) samples, and red triangles represent those having experienced a freeze phenomenon.

A discriminant analysis based on partial least squares was developed from the PCA test, showing that there were differentiable electrical responses for the samples depending on whether they had been frozen or not. The result of this PLS-DA analysis (Figure 5) show that it is possible to create a reliable model to determine the frost phenomenon in oranges using only six latent variables. For the samples used, it was possible to reach a sensitivity of 0.95 for calibration and cross-validation phases and of 0.80 for the final prediction phase.

### 3.4. ANN Results

Complementary to the preliminary PCA and PLS-DA analyses, the same data set was used to carry out a study using an ANN. To do this, different architectures and functions were considered within the nodes and, for a finer analysis, those that best suited the expected results were selected. Additionally, in order to maximize signal differences and in view of the future simplification of ANN programming selected in a microcontroller [28,39], only the first 20 data points out of 100 that the EIS device provided for each test were taken into account, corresponding to the first 20 modulus data points provided in each measurement (Figure 3).

The best ANN architecture among the studied options turned out to be a 20–9–1 structure, which means having 20 input nodes connected to a 9-node hidden layer with one final output layer. This ANN architecture gave very good results using the online back propagation function; therefore, it was the one selected to conduct the ANN modeling. Additionally, several tests using different node functions for both the hidden layer and the output node allowed us to select the hyperbolic tangent function for the hidden layer nodes and the cross-entropy function for the output layer. Once the ANN was designed, the training, validation, and test phases were carried out. 

The obtained results demonstrated that the early detection of the freeze phenomenon in the studied set of oranges by the designed ANN prediction model was possible with a high level of robustness and reliability, as the model correctly classified all the analyzed samples and consequently had a CCR = 100% for all the phases: training, validation, and test. Table 1 shows the confusion matrices for the data used in each phase and for the overall data, demonstrating the high quality of the model, since the complete data were assigned diagonally in the matrix (cells in blue in Table 1) without any classification errors.

## 4. Conclusions

The early detection of freeze damage in citrus fruits—particularly in oranges—is essential, as it helps in making decisions on what to do with the fruits when this phenomenon occurs. This process contributes to minimizing the economic consequences as well as to avoiding the introduction of fruits that may have experienced quality losses into the market.

This study proposes the use of the EIS technique by means of specific equipment and a double-needle sensor to generate electrical signals in the analyzed samples and then classify them using an ANN model. The results of this study show that it is possible to classify oranges depending on whether they have suffered a freeze phenomenon or not by using the described device and the analysis of the data via ANN. The designed model was robust, and it was reliable enough to classify all the samples tested correctly (CCR% = 100%).

This methodology surpasses the existing ones as it is easy, economical, immediate, portable, and can be used in the field. Therefore, this methodology is proposed as an alternative to the existing traditional methods for the early detection of freeze damage in oranges.

## Figures and Tables

**Table 1:** Confusion matrices for freeze damage detection by the designed artificial neural network (ANN).

Training	Validation	Test	Overall
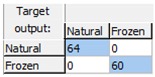	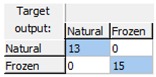	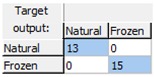	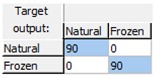

Freeze detection: Mean correct classification rate (CCR%) = 100%.
